# Cohort Analysis of ADAM8 Expression in the PDAC Tumor Stroma

**DOI:** 10.3390/jpm11020113

**Published:** 2021-02-10

**Authors:** Christian Jaworek, Yesim Verel-Yilmaz, Sarah Driesch, Sarah Ostgathe, Lena Cook, Steffen Wagner, Detlef K. Bartsch, Emily P. Slater, Jörg W. Bartsch

**Affiliations:** 1 Department of Neurosurgery, Philipps University Marburg, Baldingerstrasse, 35033 Marburg, Germany; christian@jaworek.org (C.J.); sarah.ostgathe@googlemail.com (S.O.); cookl@staff.uni-marburg.de (L.C.); 2 Department of Visceral Surgery, Philipps University Marburg, Baldingerstrasse, 35033 Marburg, Germany; yesimverel@hotmail.de (Y.V.-Y.); sarahdriesch@hotmail.de (S.D.); bartsch@med.uni-marburg.de (D.K.B.); slater@med.uni-marburg.de (E.P.S.); 3 Head and Neck Surgery, Department of Otorhinolaryngology, Justus Liebig University Giessen, Aulweg 128 (ForMED), 35392 Giessen, Germany; Steffen.Wagner@hno.med.uni-giessen.de

**Keywords:** pancreatic cancer, tumor microenvironment, tumor stroma, neutrophils, ADAM8 protease

## Abstract

Pancreatic ductal adenocarcinoma (PDAC) is a cancer type with one of the highest mortalities. The metalloprotease-disintegrin ADAM8 is highly expressed in pancreatic cancer cells and is correlated with an unfavorable patient prognosis. However, no information is available on ADAM8 expression in cells of the tumor microenvironment. We used immunohistochemistry (IHC) to describe the stromal cell types expressing ADAM8 in PDAC patients using a cohort of 72 PDAC patients. We found ADAM8 expressed significantly in macrophages (6%), natural killer cells (40%), and neutrophils (63%), which showed the highest percentage of ADAM8 expressing stromal cells. We quantified the amount of ADAM8^+^ neutrophils in post-capillary venules in PDAC sections by IHC. Notably, the amount of ADAM8^+^ neutrophils could be correlated with post-operative patient survival times. In contrast, neither the total neutrophil count in peripheral blood nor the neutrophil-to-lymphocyte ratio showed a comparable correlation. We conclude from our data that ADAM8 is, in addition to high expression levels in tumor cells, present in tumor-associated stromal macrophages, NK cells, and neutrophils and, in addition to functional implications, the ADAM8-expressing neutrophil density in post-capillary venules is a diagnostic parameter for PDAC patients when the numbers of ADAM8^+^ neutrophils are quantified.

## 1. Introduction

Pancreatic ductal adenocarcinoma (PDAC) is a highly heterogeneous tumor entity with a grim prognosis with a 5-year survival rate of less than 8% [1]. Desmoplastic reaction is very common in PDAC and accounts for a massive activation of stroma and stromal cells in the tumor microenvironment. The PDAC tumor microenvironment (TME) with its inflammatory nature activates many immune cell types in response to tumor cell derived signals (reviewed in [2]). As creators of and responders to signals in the tumor microenvironment, ADAM proteases (A disintegrin and metalloprotease) have been found to be associated with numerous functions ranging from immune cell migration and invasion [3], degradation of extracellular matrix molecules (Collagens I, IV) [4] to proteolytic inactivation of immune checkpoint inhibitors such as PD-L1 [5,6]. With their multidomain structures, ADAM proteases are capable of multiple physiological functions associated with cell adhesion, cell fusion, cell signaling, and proteolysis. Proteolysis of membrane-anchored precursor proteins by ADAMs is a key event for the generation of signaling cascades within the TME. In PDAC, a significant contribution of ADAM proteases to tumor progression was reported for ADAM8 [4], ADAM9 [7,8], ADAM10 [9], ADAM12 [10], and ADAM17 [11]. Notably, higher expression levels of these ADAM proteases were reported to be associated with a poor patient prognosis in PDAC. Similar to other solid cancers, shedding of EGF-ligands and EGFR by ADAM10 and 17 are clearly relevant for tumor signaling in pancreatic cancer [11,12]. Furthermore, there are a number of ADAM proteases lacking phenotypes in knockout mice but with a possible role in different tumor entities and specifically in PDAC, which applies for ADAM8 and ADAM9 [8]. In particular, high expression levels of ADAM8 and 9 are associated with a worsened patient prognosis [13]. In previous studies, ADAM8 in particular was described in tumor cells and functional analyses revealed a tumor-promoting effect of ADAM8 in pancreatic cancer cells [4], so that inhibition of ADAM8 in pancreatic cancer (KPC) mice using a cyclic ADAM8 inhibiting peptide (BK-1361) leads to prolonged survival and improved metrics of pathological parameters (metastasis formation, invasion of tumor cells, acinar structures). However, since ADAM8 was reported to be highly expressed in tumor-associated immune cells as shown in glioblastoma [14], the goal of the present study was to analyze the presence of ADAM8 in tumor stroma of PDAC in a cohort of 72 in-house patients.

## 2. Materials and Methods

### 2.1. Patients and Tissue Samples

A total of 72 patients with PDAC who underwent a pancreas resection in the Department of Visceral Surgery at the University Hospital Marburg were enrolled in our study (see Table 1). All tumors were histologically staged by an experienced pathologist according to UICC-TNM (Union for International Cancer Control; tumor, node, metastasis) classification 2017 [15]. All samples were obtained from the tumor bank of the Department of Pathology. Ethical approval was obtained by the local ethics committee at Marburg University, Faculty of Medicine (File Nr. 5/03). All patients provided written informed consent prior to participating in this study.

### 2.2. Immunohistochemistry (IHC)

For ADAM8 immunostaining, formalin-fixed and paraffin-embedded archived tumor samples and corresponding normal tissues were stained as follows. Paraffin sections (4 μm thickness) from PDAC patients were stained for ADAM8 using a polyclonal anti-ADAM8 antibody and a standard VectaStain Protocol. For double-staining of PDAC sections, sections were stained for ADAM8 and the respective markers for T cell markers CD3, CD4, and CD8, stellate cell marker SMA, macrophage marker CD68, natural killer cell marker CD56, and neutrophil marker myeloperoxidase (MPO). Antibodies, concentrations, and sources of primary antibodies are listed below (Table 2). Briefly, slides were heated to 60 °C for 1 h, deparaffinized using xylene, and hydrated by a graded series of ethanol washes. Antigen retrieval was accomplished by steam-heating in Target Retrieval Solution, pH9 (Agilent Dako, Waldbronn, Germany) for 30 min. For immunohistochemistry, endogenous peroxidase activity was quenched by 5 min incubation in 3% H_2_O_2_. Sections were then incubated with primary antibodies for 45 min at RT followed by biotinylated secondary antibodies for 20 min also at RT. Bound antibodies were detected using the avidin-biotin complex (ABC) peroxidase method (ABC Elite Kit; Vector Labs, Burlingame, CA, USA). Final staining was developed with the Dako DAB peroxidase substrate kit. For double staining, HRP Magenta Substrate Chromogen System was employed. Counterstaining was performed using hematoxylin. All steps following the antigen retrieval were performed using the DakoCytomation Autostainer Plus.

### 2.3. Selection of Patient Samples for Double-Staining

A total of 10 patients were selected for double-staining that reflect our total cohort by having 7 stage III (among them 2 R0) and 3 stage II samples and from these 5 patients with survival times of less than 18 months and 5 patients with survival time longer than 18 months (one patient alive with disease).

### 2.4. Quantitation

The quantitation of ADAM8-positive and marker-positive cells in paraffin-embedded and stained sections was performed using the virtual software programs Fiji Image J [16].

### 2.5. Cell Counting and Scoring of Neutrophils in PDAC Patients

Samples from 51 patients were included in neutrophil analyses and none of these patients received a neoadjuvant therapy. Planimetry measurements of three venous blood vessels on each ADAM8-stained section were performed. Later, the number of ADAM8^+^ neutrophils in the lumen of the blood vessels was scored and a ratio was calculated (cells per area). The sum of the three data sets of each patient are listed in the last column of Table A1. The blood vessels analyzed fulfilled the following criteria. The vessels were located in the center of tumor with a minimal luminal area of 2000 µm^2^. The distance between the vessels was such that three different areas of the tumor could be analyzed randomly. Blood vessels displaying fixation-related artifacts were excluded. Only cells that could be identified clearly as neutrophils with a positive staining for ADAM8 were counted.

### 2.6. Statistical Analysis

Two-way ANOVA was used for stroma cell quantifications and survival analyses. For neutrophil/survival analyses, a Pearson correlation coefficient was determined in conjunction with *t* statistics and *p*-value. Analyses were performed using Prism 6 for Mac OSX from GraphPad, San Diego, CA, USA. A value of *p* < 0.05 was considered to be significant.

## 3. Results

### 3.1. ADAM8 Expression in PDAC

**Figure 1:**
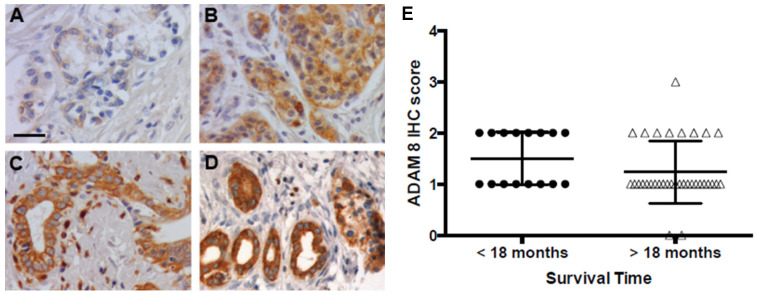
Correlation of ADAM8 staining scores with survival in patients of the PDAC cohort (*n* = 50). Four exemplary images illustrating varying levels of ADAM8 staining in patient samples. The scale bar is 25 µm. Staining intensities were determined for each section based on analysis of 5 viewing fields per section and were between 0 and 3 with no (0; (**A**)), low (1; (**B**)), moderate (2; (**C**)) and strong (3; (**D**)) ADAM8 staining. (**E**): Patients in the cohort were split into 2 groups with group 1, survival less than 18 months (•; *n* = 16) and group 2 (Δ; *n* = 34), patient survival longer than 18 months. Note that only two PDAC sections were almost negative for ADAM8. Difference is not significant.

The PDAC patient cohort (tumor, stromal cells, co-localization) consists of patients who were clinically diagnosed with PDAC in the department of visceral surgery and included in the study (see Materials and Methods section for information on exclusion criteria). From all tumor patients, paraffin-embedded sections were stained and scored for ADAM8 expression (Figure 1).

Staining intensities in tumor cells were assessed by IHC score (0–3) according to earlier studies [17] in our in house cohort. Groups were separated into two according to median survival times either shorter or longer than 18 months. No significant differences were observed between the two groups with regard to ADAM8 IHC scores.

### 3.2. Co-Localization of ADAM8 and Stromal Cell Markers in PDAC Tissue

**Figure 2:**
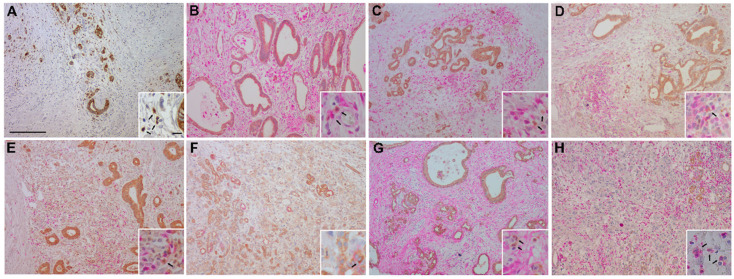
ADAM8 staining (**A**) in PDAC sections and co-localization of ADAM8 (brown) with markers (pink) for CD68 (macrophages, (**B**)), CD3 (CD3^+^ T cells, (**C**)), CD4 (CD4^+^ T cells, (**D**)), CD8 (CD8^+^ T cells, (**E**)), CD56 (NK cells, (**F**)), SMA (stellate cells, (**G**)), and MPO (neutrophils, (**H**)). In (**A**), a control stain for ADAM8 alone is shown. Bar in A, 800 μm; bar in insert (**A**), 100 μm.

In all PDAC sections stained for ADAM8, a notable expression was also observed in stromal cells (Figure 2A). To identify the stromal cell types expressing ADAM8 in PDAC, double staining of tissue with respective cell markers for T cells (CD3, CD4, CD8), natural killer (NK) cells (CD56), macrophages (CD68), neutrophils (MPO), and smooth muscle actin for stellate cells (SMA) was performed on a representative cohort of ten patients reflecting our total cohort (see Materials and Methods section for details).

We identified ADAM8-positive cells not only in the duct-like structures of the tumor area (Figure 2A), but also in the tumor microenvironment (Figure 2A–H). Stromal cells show moderate to high levels of ADAM8 staining. Significant co-staining of ADAM8 with markers for CD68 (macrophages, Figure 2B), for CD56 (NK cells, Figure 2F), and for MPO (neutrophils, Figure 2H) can be seen in Figure 2. Cells stained positively for both MPO and ADAM8 were identified to be neutrophils as evidenced by their granulocytic morphology.

### 3.3. Quantitative Analysis of Co-Localization

**Figure 3:**
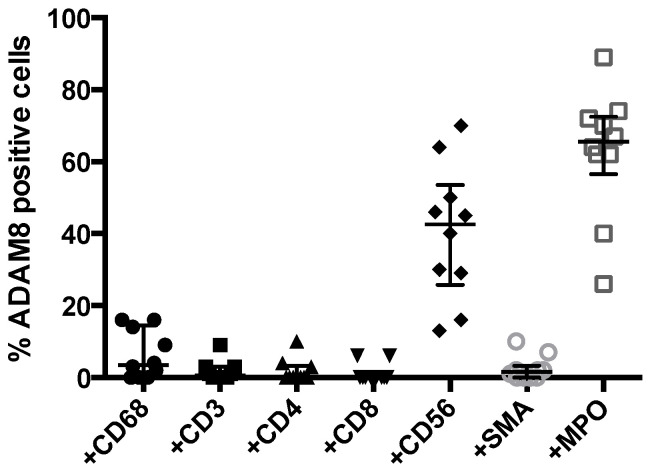
Scatter dot plot of the percentage of double-positive ADAM8/marker cells as analyzed in 10 representative PDAC sections stained for CD68 (•), CD3 (■), CD4 (▲), CD8 (▼), CD56 (♦), SMA (○) and MPO (□). Note that the frequency of ADAM8^+^ stromal cells is highest for macrophages (CD68), NK cells (CD56), and neutrophils (MPO). For each section analyzed, data are derived from quantification of 5 viewing fields in the tumor proximal stroma areas. Median values with interquartile ranges are indicated. Note that the highest frequency of co-localization of ADAM8 with stromal markers is observed for MPO (neutrophils).

ImageJ analysis on double-stained sections from a representative group of 10 patients was performed to quantify the number of specific stromal cells that were ADAM8 positive (Figure 3). Whereas T cells identified with distinct markers (CD3, CD4, and CD8) and pancreatic stellate cells (SMA) show a low percentage of co-localization, significant ADAM8-positive cell populations were observed for macrophages (CD68, 0–17%), NK cells (CD56, 18–75%), and neutrophils (MPO, 30–90%).

### 3.4. ADAM8 Expression in Neutrophils

**Figure 4:**
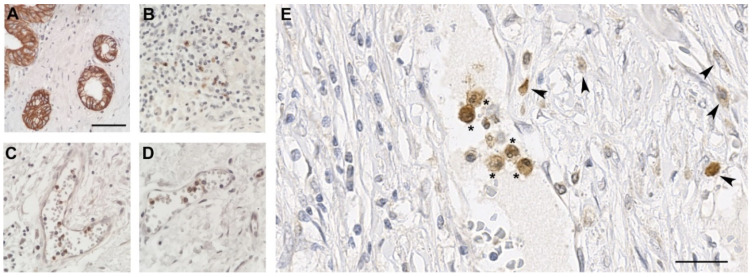
ADAM8 positive neutrophils located in tumor stromal post-capillary venules. (**A**), ADAM8 staining in duct-like structures; (**B**), ADAM8 staining in PDAC tumor stroma adjacent to duct-like structures reveals mainly ADAM8^+^ neutrophils; (**C**) and (**D**) overview venules in tumor areas with ADAM8^+^ neutrophils. (**E**), detailed view of venules with ADAM8^+^ neutrophils in vessels (asterisks) and adjacent infiltrated neutrophils (arrowheads) in a representative PDAC section. Scale bar in (**A**), valid for (**A**–**D**), 120 μm; Scale bar in (**E**), 55 μm.

We confirmed ADAM8 expression in neutrophils and their association with blood vessels in PDAC sections (Figure 3). Neutrophils enter the tissue from post-capillary venules in a process called leukodiapedesis. Thus, the likelihood of detecting neutrophils in these blood vessels is higher than in any other type of capillaries. Since post-capillary venules are large vessels, we sought to determine their frequency in PDAC sections. To obtain comparable results, neutrophils were quantified in at least 3 independent post-capillary venules with an area of >2000 μm^2^ in the core tumor tissue (Figure 4 and Table A1 in Appendix A).

**Figure 5:**
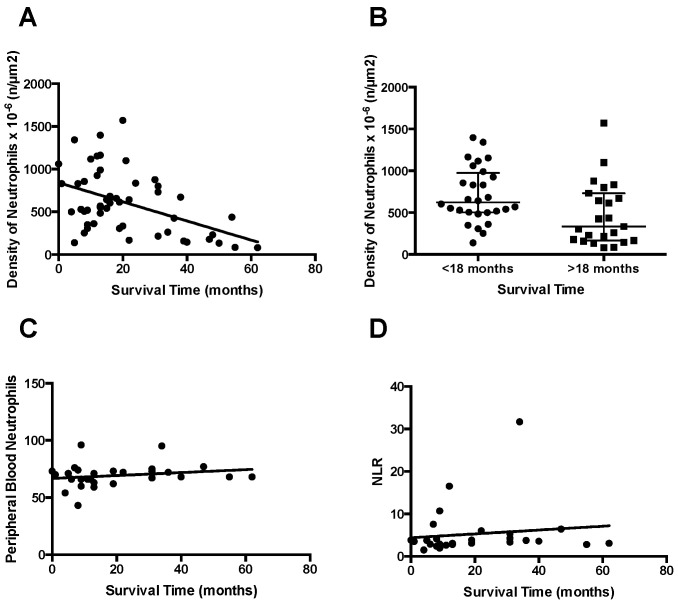
(**A**) Correlation analyses of ADAM8^+^ neutrophil counts in post-capillary venules. Each data point is the average of 3 independent post-capillary venules of an area > 2000 μm^2^ in the core tumor tissue (*n* = 50). Pearson correlation revealed r = −0.463 with *p* = 0.0006. (**B**) Neutrophil density diagram from our PDAC patient cohort split into survival time less than and greater than 18 months, *p* = 0.0128. (**C**) Neutrophil counts in peripheral blood of PDAC patients from the same cohort, *p* = 0.2797. (**D**) Neutrophil-to-lymphocyte ratio in the PDAC patient cohort, *p* = 0.5143. Note that only the ADAM8^+^ neutrophil counts in post-capillary venules are significantly correlated with PDAC patient survival.

Neutrophil numbers were correlated with patient survival data in the entire PDAC patient cohort (Figure 5A,B) where respective structures (venules) were analyzable. Moreover, we determined the neutrophil-to-leukocyte ratio (NLR) in PDAC patients where data were available and correlated these and the total blood neutrophil counts (Figure 5C,D) with survival data, respectively.

## 4. Discussion

Due to high expression levels in PDAC tumor cells, ADAM8 was previously identified as a potential therapeutic target in PDAC [4,13]. Here we confirmed earlier results in our cohort that ADAM8 is expressed in almost all PDAC samples, but no significant correlation between ADAM8 expression in tumors and survival could be drawn. Interestingly, no previous study has mentioned expression of ADAM8 in stromal cells of PDAC. Given a distinct physiological expression profile of ADAM8 in immune cells such as macrophages and leukocytes, an ADAM8 expression in stromal cells of PDAC is likely and suggests an important role of ADAM8 in the tumor microenvironment of PDAC. However, the relatively low abundance of ADAM8-positive macrophages is unexpected as under physiological conditions, e.g., in the bone marrow, macrophages are constitutively expressing ADAM8 [14,18]. In macrophages, there is experimental evidence that ADAM8 can trigger their migratory behavior into the tissue under inflammatory conditions. This has been demonstrated in muscle regeneration when ADAM8-deficient macrophages are unable to remove muscle cell debris after muscle degeneration due to lack of motility [19]. A more general effect of ADAM8 on several immune related cells was observed in allergic asthma in mice where deficiency in ADAM8 caused a significantly reduced recruitment of macrophages, neutrophils, and eosinophils to the airway inflammation site to dampen the allergic response and the asthma severity [20]. By analyzing ADAM8 expression in neutrophils, we were able to show that an increased number of ADAM8-positive neutrophils particularly in venules of the tumor areas can be of prognostic value for PDAC patients. Mechanistically, this observation could point towards a detrimental effect of ADAM8-positive neutrophils in PDAC by regulating neutrophil transmigration from the vasculature to the tumor site. A neutrophil-to-lymphocyte ratio (NLR) has been reported to be a predictive parameter in clinical studies with PDAC patients. Higher ratios have been associated with poor outcome in some studies [21,22,23]. However, the value of this ratio in terms of prognosis is controversial [24]. In addition, whereas low tumor infiltration of neutrophils has been associated with poor prognosis [25], others report neutrophil infiltration to be observed in pancreatic tumors with the poorest prognosis [26]. It is interesting to note that the number of ADAM8-positive neutrophils in venules of PDAC patients shows a better correlation than either peripheral blood neutrophil count or the NLR. Although controversial, the general findings suggest that, in pretherapy PDAC patients, the NLR is not indicative for overall survival [24], which is in accordance with our findings.

## 5. Conclusions

In PDAC, ADAM8 is significantly expressed in stromal cells, in particular in macrophages, NK cells, and neutrophils. Given the diagnostic value of neutrophil counts as reported previously [21,22,23], we propose that determination of neutrophil density in venules of tumor areas is a reliable indicator of disease progression and patient survival and could be used to stratify PDAC patients.

## Figures and Tables

**Table 1:** Clinical data on pancreatic ductal adenocarcinoma (PDAC) patient cohort used in this study (*n* = 72); abbreviations used: *: NLR: neutrophil-to-lymphocyte ratio; UICC: Union for International Cancer Control.

**Gender**	Males (%)	37 (51%)
	Females (%)	35 (49%)
**Median Age at Surgery, Years (Range)**		68 (47 to 85)
**UICC Stage, Number of Patients (%)**	I	11 (15.3%)
	II	10 (13.9%)
	III	46 (63.9%)
	IV	5 (6.9%)
**Median Survival, Months (Range)**		22 (1 to 92)
**Location**	head	65 (90%)
	body or tail	7 (10%)
Median NLR * (Range)		3.14 (1.53 to 31.67)

**Table 2:** Concentrations and sources of primary antibodies.

Antibody	Species	Working Dilution	Source
ADAM8	rabbit	1:200	R&D Systems (AF1031)
CD3	mouse	1:50	Dako (M7254)
CD56	mouse	1:10	Monosan (MON 9006)
CD68	mouse	1:200	Novus Biologicals (NB100-683)
CD163	mouse	1:50	ThermoFisher (MA5-11458)
CD4	mouse	1:100	Dako (M7310)
CD8	mouse	1:200	R&D Systems (MAB3801)
α Smooth muscle actin	mouse	1:2000	R&D Systems (MAB1420)
MPO	mouse	1:50	R&D systems (MAB3174)

## Data Availability

Patient data can be made available upon request.

## Appendix A

**Table A1:** Summarizes clinical data and neutrophil counts in venules of PDAC patients in the cohort investigated. Abbreviations used: UICC: Union for International Cancer Control.; NLR: neutrophil-to-lymphocyte ratio.

Number	Gender	Age at Surgery (Years)	UICC Stage	Survival (Months)	Location	NLR	Density of Neutrophils (n/µm^2^)
1	f	71	II	92	head	4.60	
2	m	85	IV	20	head	n.a.	334 × 10^6^
3	f	75	III	11	head	2.64	360 × 10^6^
4	m	65	III	31	head	4.29	216 × 10^6^
5	m	74	III	30	head	n.a.	878 × 10^6^
6	m	61	III	55	head	2.83	84 × 10^6^
7	m	56	III	62	head	3.09	81 × 10^6^
8	f	55	III	19	head	3.84	305 × 10^6^
9	m	73	III	6	body	2.87	831 × 10^6^
10	m	49	II	8	head	2.39	856 × 10^6^
11	m	67	III	5	head	n.a.	139 × 10^6^
12	f	51	III	40	head	3.58	146 × 10^6^
13	m	68	III	50	head	n.a.	134 × 10^6^
14	w	77	II	34	head	31.67	260 × 10^6^
15	f	85	II	0	head	3.84	1061 × 10^6^
16	m	52	IV	16	body	n.a.	603 × 10^6^
17	f	68	III	20	tail	n.a.	1573 × 10^6^
18	f	82	III	22	head	6.00	643 × 10^6^
19	f	53	III	35	head	n.a.	
20	f	69	IV	15	body	n.a.	540 × 10^6^
21	f	74	III	4	head	1.53	501 × 10^6^
22	m	68	III	4	head	n.a.	
23	f	78	III	39	head	n.a.	159 × 10^6^
24	m	56	III	30	head	n.a.	
25	f	65	III	38	head	n.a.	672 × 10^6^
26	f	50	III	13	head	2.81	484 × 10^6^
27	f	62	I	42	head	n.a.	
28	m	78	III	31	head	3.35	800 × 10^6^
29	f	75	I	32	head	n.a.	
30	m	68	I	1	head	3.50	831 × 10^6^
31	m	72	III	36	head	n.a.	
32	m	66	II	33	head	n.a.	
33	m	78	I	28	head	n.a.	
34	f	75	I	35	head	6.83	
35	f	79	III	22	head	n.a.	168 × 10^6^
36	m	60	III	9	head	2.87	519 × 10^6^
37	f	60	I	33	head	2.75	
38	f	58	III	27	head	2.79	
39	f	57	III	19	head	3.10	616 × 10^6^
40	m	67	III	28	head	n.a.	
41	m	64	III	24	head	n.a.	836 × 10^6^
42	f	68	I	29	head	n.a.	
43	m	60	I	n.a.	head	n.a.	
44	m	60	III	28	head	2.87	
45	f	51	III	16	head	n.a.	682 × 10^6^
46	f	78	III	28	head	2.00	
47	f	79	I	26	head	n.a.	
48	m	47	III	10	head	n.a.	1117 × 10^6^
49	m	65	II	12	body	16.50	1154 × 10^6^
50	f	79	III	13	head	2.86	569 × 10^6^
51	m	72	III	13	head	2.86	1398 × 10^6^
52	m	58	III	48	head	n.a.	230 × 10^6^
53	f	71	II	75	head	2.67	
54	m	78	III	12	head	n.a.	926 × 10^6^
55	m	64	III	54	head	n.a.	438 × 10^6^
56	m	70	I	36	body	3.79	425 × 10^6^
57	f	61	III	31	head	5.36	733 × 10^6^
58	m	71	III	13	head	n.a.	1164 × 10^6^
59	m	68	III	21	head	n.a.	1099 × 10^6^
60	m	75	II	47	head	6.42	179 × 10^6^
61	f	77	III	57	head	3.14	
62	f	75	III	9	head	1.94	349 × 10^6^
63	m	80	II	8	head	4.11	252 × 10^6^
64	m	52	III	13	head	n.a.	991 × 10^6^
65	f	78	III	7	head	7.60	529 × 10^6^
66	m	80	II	9	head	10.67	308 × 10^6^
67	m	67	III	15	head	n.a.	643 × 10^6^
68	f	64	IV	13	body	3.09	553 × 10^6^
69	f	68	IV	18	head	n.a.	658 × 10^6^
70	f	73	III	5	head	3.74	1342 × 10^6^
71	f	72	III	8	head	n.a.	506 × 10^6^
72	m	77	I	16	head	n.a.	
